# Impact of "+Contigo" training on the knowledge and attitudes of health care professionals about suicide

**DOI:** 10.1590/0104-1169.3503.2467

**Published:** 2014

**Authors:** José Carlos Santos, Rosa Maria Pereira Simões, Maria Pedro Queiroz de Azevedo Erse, Jorge Daniel Neto Façanha, Lúcia Amélia Fernandes Alves Marques

**Affiliations:** 1 PhD, Adjunct Professor, Escola Superior de Enfermagem de Coimbra, Coimbra, Portugal; 2 MSc, RN, Casa de Saúde Rainha Santa Isabel, Instituto das Irmãs Hospitaleiras do Sagrado Coração de Jesus, Coimbra, Portugal; 3 Specialist in Mental Health Nursing and Psychiatry, RN, Centro Hospitalar e Universitário de Coimbra, Coimbra, Portugal; 4 Specialist in Community Nursing, RN, Departamento de Saúde Pública, Administração Regional de Saúde do Centro, IP, Coimbra, Portugal

**Keywords:** Suicide/prevention & control, Training, Health Knowledge, Attitudes, Practice, Health Personnel

## Abstract

**OBJECTIVES:**

to evaluate the results of "*+Contigo*" training, developed by nurses and directed at 66 health professionals of integrated school health teams in Primary Health Care.

**METHOD:**

quantitative with data collection through the Suicide Behavior Attitude Questionnaire, administered before and after the training.

**RESULTS:**

significant increases were observed in suicide prevention knowledge and in changing attitudes of health professionals towards individuals with suicidal behavior.

**CONCLUSION:**

these results allow us to affirm that nurses hold scientific and pedagogical knowledge that grant them a privileged position in the health teams, to develop training aimed at health professionals involved in suicide prevention.

## Introduction

Suicide is a complex, multifaceted, underestimated and often avoidable phenomenon. In Portugal, about 1000 suicides are committed per year, with an upward trend since the year 2000. In the central region, suicide rates have slightly increased in recent years (11.2/100,000, in 2009), superior to the national rate (9.5) for the same period. A higher incidence level is found in the South of Santarém and suicide is characterized as a phenomenon mainly of elderly people. Parasuicidal behaviors, however, mainly involve young people, with a uniform distribution at the national level.

Scientific evidence proves that suicide is significantly associated with mental illness, based on which it can be affirmed that an effective treatment of mental illness increases the probability of reducing suicide risks. For this treatment to be effective, however, the correct and timely identification of the individuals at risk and the elimination of access barriers to specialized care are fundamental. These barriers include, among others, the stigma of mental illness that still exists, even among health professionals, and the lack of investments in health professionals' education^(^ 1 ^)^.

The interpretation of suicidal behaviors by health professionals is very important to determine their actions involving these individuals, concerning hospital care and forwarding^(^ 2 ^)^. In that sense, countless studies have focused on the prevalent attitudes of health professionals towards people with suicidal behaviors, suggesting that negative attitudes, criticism and devaluation are predominant among these professionals^(^ 3 ^-^ 7 ^)^.

Feelings like anxiety and insecurity, fear, excessive involvement, sympathy, irritation and anger also prevail^(^ 8 ^)^. Knowledge on the phenomenon is one of the variables studies, about which a relative consensus exists that it is insufficient^(^ 4 ^-^ 5 ^)^.

The comparison between health professionals' attitudes towards people with suicidal behaviors and people with physical illness shows the professionals' hostile attitudes towards the former^(^ 9 ^)^.

As to the differences found in the health professionals' attitudes per professional category, physicians generally show more negative attitudes than nurses^(^ 10 ^)^. Psychiatrists stand out because their attitudes are more positive than of physicians in other specialties^(^ 11 ^)^. In a study developed in the United Kingdom, however, the physicians were more aware of the suicide risk than the nurses^(^ 4 ^)^.

The need for permanent education and technical-social skills training in suicidology has been widely emphasized and defended as fundamental orientations to prevent suicide, to be systematically disseminated in the community and emergency context^(^ 3 ^,^ 7 ^,^ 12 ^-^ 18 ^)^. Parallel to these orientations are the results obtained in educational interventions, which are mostly stimulating, as verified in the literature. Based on the analysis of these results, it can be concluded that educational interventions are effective to improve knowledge, change attitudes and improve the health professionals' competency levels to prevent suicide^(^ 3 ^-^ 4 ^,^ 7 ^,^ 19 ^)^. These effects were mostly assessed in the short term and no differences were verified between general and psychiatric hospitals^(^ 3 ^,^ 11 ^,^ 13 ^,^ 20 ^)^.

After an educational intervention, health professionals were capable of applying the new knowledge in clinical practice, considering themselves more aware of the problem and more competent in suicide risk management. These professionals indicated the existence of unprepared professionals to deal with the problem, the lack of support from expert professionals and the absence of guidelines for good practices as barriers^(^ 16 ^)^.

Based on the abovementioned aspects, it is considered that health professionals' education should address self-knowledge, knowledge, understanding, attitudes, communication and suicidal behaviors. Besides health professionals' formal education, in the different suicide prevention contexts, Portuguese and international orientations are also considered pertinent, such as the *Guia Orientador de Boas Práticas para a Prevenção de Sintomatologia Depressiva e Comportamentos da Esfera Suicidária* [Guide of Good Practices to Prevent Depressive Symptoms and Suicidal Behaviors], published by the Portuguese Order of Nurses^(^ 21 ^)^.

The question about how long the changes in the professionals' behavior towards people with suicidal behaviors will continue remains unanswered.

Based on these considerations and in the context of the Suicide Prevention Project in the School Context "*+Contigo*", designed and implemented by the authors of this paper, which recommends the participation of all stakeholders in the school context, training courses were developed for Primary Healthcare professionals.

## Method

A quasi-experimental study without a control group was developed, with the following objectives:

-to assess the results of training directed at Primary Healthcare professionals;

-to verify whether knowledge and attitude differences exist with regard to suicidal behavior in function of the variables age, gender, experience and function.

The impact of the training is assessed through the analysis of the following aspects: attitudes towards the depressed patient, negative feelings towards individuals with suicidal behavior, perceived professional skills, right to suicide and knowledge in suicide prevention^(^ 2 ^)^.

The training was offered during three courses of 21 hours each, aimed at Primary Healthcare professionals and more specifically school health team professionals. All courses were promoted by the training division of the Regional Health Administration of the Central Region, working in close articulation with the coordinating team of the project "*+Contigo*", who served as the trainers in all courses taught. It should be highlighted that these trainers possess a specialized educational background in Mental Health and Psychiatry with emphasis on the theme suicide and experience in pairing^(^ 7 ^,^ 21 ^)^.

The training course "*+Contigo*" is organized around three thematic axes: adolescence, depression and suicidal behaviors. During the course, besides concepts and existing evidence about the theme, debate and discussion are encouraged about myths, practices and the operation of the project in the educative community, particularly in the school context.

Concerning the sample, the training courses "*+Contigo*" included 66 professionals from the school health teams of ten Health Center Groups of the Regional Health Administration in the Central Region. These professionals were selected based on inclusion criteria the "*+Contigo*" project coordination team had set in partnership with the Regional Health Administration in the Central Region, after presenting the project to all professionals responsible for all Health Center Groups in the defined region.

The inclusion criteria are: motivation of the health professionals to invest in the suicide prevention problem and attending the training module together with the project coordination team; the professionals responsible for the school should manifest their desire to participate and candidate themselves together with the school health team; taking part in the education program for school health and the activity plan of the School Health team; and agreement of the professionals in charge of education to participate.

The data collection instrument included a sociodemographic data form (gender, age, function and experience) and the adapted Suicide Behavior Attitude Questionnaire^(^ 2 ^)^. The professionals in the sample completed these instruments at the start and end of the training program "*+Contigo*". Before distributing the questionnaires, the professionals were asked to participate voluntarily and received information about the voluntary nature of their participation, besides guarantees of anonymity and confidentiality.

The Suicide Behavior Attitude Questionnaire consists of 25 assertions, scored between one and five, is self-completed and measures the professionals' attitudes, considering cognitive, affective and behavioral aspects. The higher the global score, the greater the change will be in terms of knowledge and attitudes.

In the constitution of the Suicide Behavior Attitude Questionnaire, eight assertions were grouped in three factors to facilitate the assessment of the attitude change, which are: Factor 1 (F1) - negative feelings towards individuals with suicidal behavior, Factor 2 (F2) - perceived professional skills and Factor 3 (F3) - the right to suicide. The other assertions relate to knowledge about suicide prevention (13 assertions) and the professionals' attitudes towards depressed individuals (4 assertions).

## Results

Sixty-six Primary Healthcare professionals participated in the study. The distribution according to sociodemographic characteristics shows that the majority is female (92.40%). Concerning the function, the large majority are nurses (84.80%), in accordance with the inclusion criteria. As regards the age variable, the mean age of the sample elements is 41.53, with a minimum age of 26 and a maximum of 61 years. The length of experience ranges between one and 34 years, with a mean 17.14 years and a standard deviation of 7.91 years.

The application of the Suicide Behavior Attitude Questionnaire shows that the global score before the course (initial application of the questionnaire) corresponded to 3.40, increasing to 3.92 in the financial assessment, after the training course. This result derives from higher mean scores on 22 questionnaire items. The difference of means found was statistically significant on nine items scored after the application of Student's t-test for independent samples.

Concerning the factors assessed in the Suicide Behavior Attitude Questionnaire, higher means were found in four of these factors, three of which with statistically significant differences: attitudes towards depressed individuals, perceived professional skills and knowledge on suicide prevention (p<0.05). It should be highlighted that the only factor in which no increase was found between the mean scores on the initial assessment "before the course" and the final assessment "after the course" was factor 3, called the right to suicide.

Based on the statistical analysis, it can be added that no statistically significant differences were verified in the attitudes towards suicidal behavior in function of the variables gender, function performed, age and length of experience.

## Discussion

The non-probabilistic sampling method is considered a methodological limitation, as the inclusion criteria, like the participation in the school health team and the personal and team motivation conditioned to the selection of an intentional sample. This type of sampling seemed inevitable, given the need to prepare these professionals to constitute the local "*+Contigo*" team and to be able to implement the Suicide Prevention Project in the School Context "*+Contigo*".

Another methodological limitation is the absence of a control group and of follow-up. These were conscious options, keeping in mind that all health professionals who attended the training course "*+Contigo*" adhered to the project and, consequently, closely articulated with and were followed by the regional "*+Contigo*" team. This fact can interfere in the training impact and the long-term assessment. These options were based on studies that concluded that skills development after training is statistically significant in comparison with the control group and that the competences are mostly maintained six months after the training^(^ 3 ^,^ 5 ^,^ 11 ^)^.

Concerning the discussion of the results found, it can be affirmed that, at the level of the Suicide Behavior Attitude Questionnaire, an increase was verified in the mean scores for 22 out of 25 questionnaire items, with statistically significant differences in nine items. The analysis of these items grouped per factors showed higher mean scores in four of these factors and statistically significant differences in three.

The comparison between the results obtained in our study with those of another study^(^ 22 ^)^ in which the Suicide Behavior Attitude Questionnaire was also used showed a significant variation after the training in 18 out of 25 questionnaire items used. These authors also concluded that the nurses express greater professional skills after the training^(^ 19 ^)^.

Some studies showed a 44% increase in acceptable levels when compared to the pre-training levels^(^ 23 ^)^, higher than the levels found in this study, which showed a global increase by 10.4% in the total number of items in the Suicide Behavior Attitude Questionnaire.

In a study of the professional skills of elements in the educative community, in the school context, it was verified that, after receiving gatekeeper training, these professionals referred less students to specialized monitoring or mental health services when compared to the professionals who did not receive gatekeeper training^(^ 23 ^)^. Team training increased the proportion of people who intervened in people with suicide risks by 20%.

According to our results, it has been concluded earlier that programs for community members demonstrated positive changes in the knowledge and attitudes towards suicide^(^ 23 ^)^. More specifically regarding the professionals' attitudes and using an attitude inventory before and after formal training, a reduction was found in the biased or negative attitudes expressed^(^ 3 ^-^ 4 ^)^ and significant improvements in the participants' attitudes and confidence^(^ 14 ^)^.

Furthermore, in the impact analysis of a brief intervention program on suicide prevention and health professionals' attitudes towards suicide, it was conclude that the brief training was determinant to provide the health professionals with knowledge about suicide prevention, independently of their clinical experience, allowing them to improve their short-term attitudes and beliefs on suicidal behaviors^(^^24^^)^.

The analysis of the mean scores obtained for the factors under investigation showed that the "right to suicide" was the only factor that did not show a higher mean score, with two assertions whose means dropped from 2.00 and 4.50 (before the training course) to 1.39 and 4.22, respectively (after the training course). This factor was a source of great discussion among the professionals who developed the training course as, if suicide can be accepted in moral and philosophical terms, that is, it is considered that people are entitled to kill themselves, in legal terms, the Portuguese law punishes whoever encourages another person towards suicide (art. 135 of the Penal Code) and, in religious terms, almost all religions seriously condemn suicide, and the Catholic Church prohibits people with suicidal behaviors from receiving the holy orders. The fact that 79.5% of the Portuguese people are Catholic illustrates the results found.

In the analysis of this matter, it was concluded that the belief that a person is not entitled to commit suicide was stronger among older professionals, professionals who did not have contact with people with suicidal behaviors, professionals with a family history of suicide, protestant professionals and those who used to attend more religious services^(^^24^^)^.

The analysis of the sociodemographic variables gender, function performed, age and length of experience showed that there are no statistically significant differences in knowledge and attitudes towards suicidal behavior. Some studies demonstrate that health professionals, independently of their clinical origin and previous work experience, present similar scores in terms of their attitudes and beliefs regarding the suicide phenomenon^(^ 17 ^,^^24^^)^. In most of the studies analyzed, physicians generally present more negative attitudes than nurses^(^ 10 ^)^. The reason why this was not verified may be due to the small number of non-nurses.

The results regarding the gender variable were in accordance with the studies analyzed, that is, no knowledge and attitude differences are verified in function of gender.

Concerning the function performed and although there are various studies that show statistically significant differences, such a limited number of non-nursing elements in the sample (physicians, psychologists and social workers) does not permit any inferences.

## Conclusion

Based on the strong evidence of negative attitudes towards individuals with suicidal behaviors, it can be affirmed that researchers should further invest in the suicidology area and, hence, that more educative interventions should be offered to all stakeholders in the management of the phenomenon.

In this study, the impact of the training was assessed in terms of changes in Primary Healthcare professionals' knowledge and changes in attitudes towards suicidal behavior. It was concluded that this training was effective to promote the desired changes in the participants, from the perspective of suicide prevention knowledge as well as attitude changes. The changes that occurred may contribute to the early detection of people at risk of suicide and to improve their forwarding for appropriate treatment/monitoring.

In view of these results, it can be affirmed that education and formal training should be offered to all health professionals involved in suicide prevention and in the treatment of people with this kind of behavior. Nurses possess scientific and pedagogical knowledge that grant them a privileged position in the health teams to develop this education.

It is emphasized that, besides the formal education of health professionals, and more specifically the nurses who work in the different suicide prevention contexts, it is also fundamental to use and apply the orientations in the *Guias Orientadores de Boas Práticas para a Prevenção de Sintomatologia Depressiva e Comportamentos da Esfera Suicidária* [Guide of Good Practices to Prevent Depressive Symptoms and Suicidal Behaviors] in clinical practice.
